# Hybrid De Novo Whole-Genome Assembly, Annotation, and Identification of Secondary Metabolite Gene Clusters in the Ex-Type Strain of *Chrysosporium keratinophilum*

**DOI:** 10.3390/jof9040389

**Published:** 2023-03-23

**Authors:** Alan Omar Granados-Casas, Angie Paola Sastoque, Alberto Miguel Stchigel, Ana Fernández-Bravo, José Francisco Cano-Lira

**Affiliations:** Mycology Unit, Medical School, Universitat Rovira i Virgili, C/Sant Llorenç 21, 43201 Reus, Spain

**Keywords:** ANI, Ascomycota, biosynthetic pathways, *Chrysosporium keratinophilum*, genome, Onygenales

## Abstract

*Chrysosporium* is a polyphyletic genus belonging (mostly) to different families of the order Onygenales (Eurotiomycetes, Ascomycota). Certain species, such as *Chrysosporium keratinophilum*, are pathogenic for animals, including humans, but are also a source of proteolytic enzymes (mainly keratinases) potentially useful in bioremediation. However, only a few studies have been published regarding bioactive compounds, of which the production is mostly unpredictable due to the absence of high-quality genomic sequences. During the development of our study, the genome of the ex-type strain of *Chrysosporium keratinophilum*, CBS 104.66, was sequenced and assembled using a hybrid method. The results showed a high-quality genome of 25.4 Mbp in size spread across 25 contigs, with an N50 of 2.0 Mb, 34,824 coding sequences, 8002 protein sequences, 166 tRNAs, and 24 rRNAs. The functional annotation of the predicted proteins was performed using InterProScan, and the KEGG pathway mapping using BlastKOALA. The results identified a total of 3529 protein families and 856 superfamilies, which were classified into six levels and 23 KEGG categories. Subsequently, using DIAMOND, we identified 83 pathogen–host interactions (PHI) and 421 carbohydrate-active enzymes (CAZymes). Finally, the analysis using AntiSMASH showed that this strain has a total of 27 biosynthesis gene clusters (BGCs), suggesting that it has a great potential to produce a wide variety of secondary metabolites. This genomic information provides new knowledge that allows for a deeper understanding of the biology of *C. keratinophilum*, and offers valuable new information for further investigations of the *Chrysosporium* species and the order Onygenales.

## 1. Introduction

The genus *Chrysosporium* was proposed by Corda to introduce a single species, *Chrysosporium corii* [1]. However, Saccardo [2] synonymized that genus with *Sporotrichum* and, consequently, the former fell into oblivion. More than fifty years later, Hughes [3] reintroduced *Chrysosporium* for *C. corii* and *Chrysosporium pannorum* (syn. *Geomyces pannorum*), restricting the generic concept of *Sporotrichum* to those species with wide hyphae, dark conidia and the absence of intercalary conidia. In a revision carried out by Carmichael [4], *Blastomyces*, *Emmonsia*, *Geomyces*, *Myceliophthora,* and *Zymonema* were synonymized with *Chrysosporium*, leaving that genus morphologically highly one-sided. Dominik [5] expanded Carmichael’s concept of *Chrysosporium* a little more, including *Sepedonium*, a genus that, like *Sporotrichum*, was later demonstrated to have phylogenetic links with basidiomycetous fungi [6]. Van Oorschot [6], in her monograph on *Chrysosporium* and allied genera, restored the order to the genus, disaggregating *Emmonsia*, *Geomyces*, *Myceliophthora,* and *Zymonema* from them, and introducing the genus *Trichosporiella*, based on colony features, conidial morphology, temperature resistance, and keratin degradation, among other phenotypic characters. Van Oorschot [6] also remarked the connection between the species of *Chrysosporium* with ascomycetous sexual morphs belonging to the order Gymnoascales (Ascosphaeraceae, Gymnoascaceae and Onygenaceae) and the family Sordariaceae [7]. These species of *Chrysosporium* are characterized by the production of hyaline, septate hyphae and mostly one-celled, hyaline or brightly colored, small, subglobose-to-pyriform or claviform conidia. These conidia are born singly (rarely in short chains), laterally and terminally (holoblastic ontogeny) on the fertile hyphae, and/or intercalary (holothallic ontogeny) along the hyphae [8,9].

The phylogenetic study performed by Vidal et al. [10], based on the analysis of the nucleotide sequences of the ITS region, demonstrated that the genus *Chrysosporium* is highly polyphyletic, since it is possible to find some of their species scattered among different orders and families of the phylum Ascomycota. In fact, most fungal taxonomists agree with Vidal et al. [10] that this genus should be restricted to species in the order Onygenales. Currently, according to the Mycobank, this genus contains over 121 accepted species (https://www.mycobank.org); (accessed 24 January 2023).

*Chrysosporium* species are opportunistic pathogens, with importance to the biotechnology and pharmaceutical fields. Most reports of *Chrysosporium* spp. as etiologic agents are associated with infections of the nails, skin, hair scalp and, to a lesser extent, with pulmonary diseases. However, these reports must be treated with care, since in most cases, the causative agent has not been correctly (molecularly) identified. Additionally, there is no broad consensus among clinical mycologists about the pathogenic potential of these species [11,12,13,14].

Regarding the potential use in biotechnology, many enzymes have been reported to be produced by *Chrysosporium* spp., such as N-acetylglucosaminidase, amylase, chymotrypsin, cystine-aryl-amidase, elastase, esterase-lipase, α-fucosidase, α-galactosidase, β-galactosidase, α-glucosidase, β-glucosidase, keratinase, leucine-aryl-amidase, α-mannosidase, pectinase, urease, α-glucosidase and urease, among others [15,16,17]. The genus *Chrysosporium* has grabbed the attention of researchers due to its ability to produce keratinases, enzymes which act by hydrolyzing keratin, the main protein in skin, hairs, nails, hooves and feathers, making it a potential bioremediation for decomposing such structures in wastewater and solid waste [18,19,20].

Only a few genomes of the *Chrysosporium* spp. are available in databases, and studies on the production of bioactive compounds by these sorts of fungi are scarce. In the present study, therefore, we sequenced and re-built the genome of the ex-type strain of *Chrysosporium keratinophilum*, with a combination of Illumina and PacBio sequencing strategy, followed by functional annotation. *Chrysosporium keratinophilum* (ex-type strain CBS 104.62) was isolated from a soil sample in Papua New Guinea in 1957 [21], and connected with their sexual morph, *Aphanoascus keratinophilus*, in 1990 [22]. Moreover, to test the potential secondary metabolite biosynthetic pathways of this strain, an AntiSMASH analysis was carried out. This resource provided a solid basis for performing comparative genomics and widened our understanding of the genus *Chrysosporium.*

## 2. Materials and Methods

### 2.1. DNA Extraction, Sequencing and Assembling

The genomic DNA of the ex-type strain of *Chrysosporium keratinophilum*, CBS 104.62, was extracted using the modified DNeasy^®^ Plant Mini Kit protocol (Qiagen, Hilden, Germany). The DNA quality was checked by Nanodrop 2000 (Thermo Scientific, Madrid, Spain) and Qubit 2.0 Fluorometer (Invitrogen, Carlsbad, CA, USA). The extracted DNA was sequenced by Macrogen (Seoul, Korea) using short and long reads, with the use of two sequencing platforms, Illumina NovaSeq6000 (Illumina, San Diego, CA, USA) 150 PE (150 × 2 bp) and Pacbio Sequel I (Pacific Biosciences of California, Inc., Melon Park, CA, USA). For Illumina sequencing, the library was constructed using the Truseq Nano DNA library, for Pacbio sequencing a 10-kb insert library was constructed using SMARTbell Express. The raw Illumina reads were visualized using the FastQC v0.11.9 [23] tool to verify the quality of the reads and identify low-quality reads. Later, the adapter sequences and the low-quality reads were removed by Trimmomatic v0.39 [24], using the ILLUMINACLIP, SLIDINGWINDOW and MINLEN options. Hybrid assemblies with short and long reads were performed using the SPAdes v3.13.0 [25] and MaSuRCA v4.0.5 [26] software, with default settings. All assemblies obtained were evaluated using QUAST v5.1.0rc.1 [27] and BUSCO v5.3.1 [28] to assess the completeness of the genome. Based on QUAST and BUSCO results, only one assembly was considered for downstream analysis. The best result draft assembly was polished using Illumina short-read data with POLCA (from MaSuRCA v.4.0.5).

### 2.2. Genomic Indexes

To verify their taxonomic identity, seven complete genomes of phylogenetically close related taxa of the order Onygenales were downloaded from the GenBank Genome database of the NCBI (https://www.ncbi.nlm.nih.gov/genome (accessed on 22 April 2022)): *Amauroascus niger* (asexual morph unknown), *Aphanoascus verrucosus* (asexual morph *Chrysosporium tropicum*), *Brunneospora queenslandica* (asexual morph *Chrysosporium queenslandicum*), *Coccidioides immitis* (asexual morph malbranchea-like), *Coccidioides posadasii* (asexual morph malbranchea-like), *Ophidiomyces ophidiicola* (asexual morph chrysosporium-like) and *Uncinocarpus reesii* (asexual morph unknown) (Table 1). Later, an average nucleotide identity (ANI) analysis was carried out using Pyani v0.2.11 with ANIb (BLAST+) [29].

### 2.3. Genome Prediction and Annotation of the Strain CBS 104.62

Barrnap v0.9 [30] and tRNAscan-SE v2.0.9 [31] were used to predict rRNAs and tRNAs, respectively. The annotation of the genome was performed using BRAKER2 v2.1.6 [32] pipeline, along with GeneMark-ET and AUGUSTUS packages. Subsequently, functional annotation was carried out using InterProScan v5.55-88.0 [33], to determine the incidence of protein families and superfamilies in the genome. Finally, functional annotation of *C. keratinophilum* CBS 104.62 was performed using similarity searches against the KEGG (Kyoto Encyclopedia of Genes and Genomes) database, using the BLASTKOALA v2.2 (https://www.kegg.jp/blastkoala/ (accessed on 5 July 2022)) annotation web server. The carbohydrate-active enzymes (CAZymes) were determined running Run_dbCAN v3 [34], using DIAMOND, with default settings. The pathogenicity-related genes were identified using DIAMOND v2.0.15 [35] against the pathogen–host interaction (PHI) [36] database, using BLASTp, with parameters of e-value of 1 × 10^−5^, max-target sequence alignment 1, 80% identity, amino acid length ≥ 100, 60% query coverage and 60% subject coverage. Finally, biosynthesis gene clusters (BGCs) were predicted using AntiSMASH v6.1.1 [37], with default settings.

## 3. Results and Discussion

### 3.1. Genome Information and Comparison with the Closest Species

**Figure 1:**
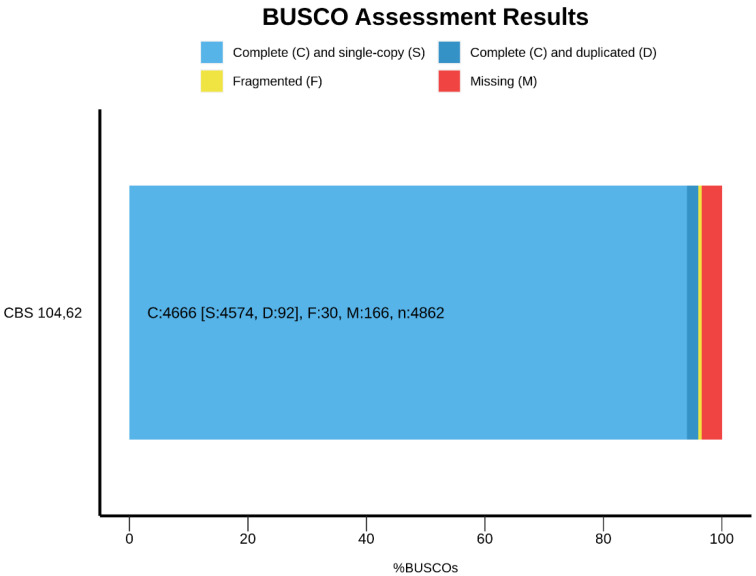
The BUSCO (v.5) report for the final hybrid assembly of an ex-type strain (CBS 104.62) of *Chrysosporium keratinophilum* genome. The light blue portion of the bar represents complete (C) and single-copy (S) orthologs, dark blue represents complete and duplicated (D) orthologs, yellow represents fragmented (F) BUSCO genes and red represents missing (M) BUSCO genes.

We present the first hybrid de novo genome sequencing of the ex-type strain of *Chrysosporium keratinophilum* using short- and long-read technologies. The QUAST analysis showed that the best assembly was obtained with MaSuRCA. The resulting polished genome consisted of 25.4 Mbp, spread across 25 contigs with an N50 of 2.0 Mb and a BUSCO score of 96.0%. This last result is comparable to the *C. immitis* RS, *C. posadasii* C735 delta SOWgp and *A. verrucosus* IHEM 4434 genome assemblies (96.8%, 96.8% and 96.3%, respectively), indicating that our assembly was relatively contiguous (Figure 1).

A total of 166 tRNAs, with a length ranging from 67 bp to 129 bp, and 24 rRNAs were predicted in the genome.

Assembly statistics of *Chrysosporium keratinophilum*, with its closest phylogenetically related species, are referred to in Table 2.

### 3.2. Average Nucleotide Identity

**Figure 2:**
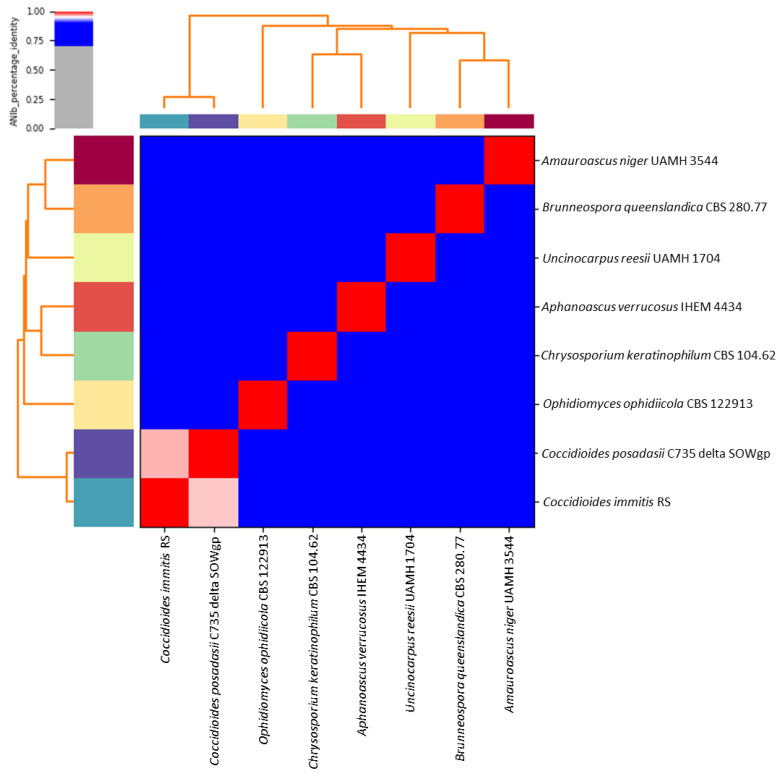
Heatmap generated showing average nucleotide identities (ANIs) with Pyani. Genome comparison of the draft genome of *Chrysosporium keratinophilum* CBS 104.62 and other Onygenales species. The heatmap shows the level of similarity, whereby the red color (100%, 1.0 ident.) gives high similarity and gray color (70%, 0.7 ident.) shows low similarity.

Based on the whole-genome alignment, the average nucleotide identity (ANI) showed values between some members of the Onygenales from 96.14 to 72.48%. These results confirmed that *Chrysosporium keratinophilum* belongs to the family Onygenaceae, showing a close relationship with *Aphanoascus verrucosus* IHEM 4434, with an ANI value of 81.19%, although it is loosely related to other species that are more closely related to the Onygenales (*Amauroascus niger* UAMH 3544, *Brunneospora queenslandica* CBS 280.77, *Coccidioides immitis* RS, *Coccidioides posadasii* C735 delta SOWgp, *Ophidiomyces ophiodiicola* CBS 122,913 and *Uncinocarpus reesii* UAMH 1704) (Figure 2). Based on the ANI results, we accept *Chrysosporium keratinophilum* CBS 104.62 as belonging to the genus *Aphanoascus*, as has been previously proposed [22].

In the present study, the highest ANI value obtained was between *Coccidioides immitis* RS and *Coccidioides posadasii* C735 delta SOWgp (ANI value = 96.1%), and the lower values were shown by *Ophidiomyces ophiodiicola* CBS 122,913 when it was compared with the other analyzed strains (ANI values ≤ 72.7%). *Brunneospora queenslandica* CBS 280.77 and *Amauroascus niger* UAMH 3544 showed an ANI value of 83.43%. Our results suggest this ANI value is too high for two strains belonging to different genera, because previous studies have obtained ANI values close to 79% for fungi of the same genus [38]. Therefore, an exhaustive taxonomic review of the Onygenales is recommended in order to look for possible errors in the taxonomic assignment or for limitations of the ANIs in discriminating between the genera of that order.

### 3.3. Prediction of Genes from the Assembled Genome

Gene annotation, using BRAKER2 pipeline, resulted in 34,824 coding sequences (CDS) and 8002 protein sequences. Functional annotation, using Interproscan with Pfam and SUPERFAMILY options, produced a total of 3529 protein families and 856 superfamilies as the results (Supplementary Tables S1–S4). Annotations based on the Pfam and SUPERFAMILY databases assigned functions to 76.6% and 62.7%of the predicted proteins, respectively. The most prevalent Pfam dominants included WD domain G-beta repeat, protein kinase domain, reverse transcriptase (RNA-dependent DNA polymerase), Ankyrin repeats (three copies), and mitochondrial carrier protein as the most prevalent families. In the case of superfamilies, the analysis showed that the five most prevalent were: a P-loop containing nucleoside triphosphate hydrolases, protein kinase-like (PK-like), ribonuclease H-like, NAD(P)-binding Rossmann-fold domains and DNA/RNA polymerases.

Previous studies have shown a fluctuating number of gene families in some members of the Onygenales [39,40,41]. The genome analysis of *C. keratinophilum* showed a reduction in the number or an absence of gene families related to the degradation of the plant cell wall, such as the cellulase (glycosyl hydrolase family 5), fungal cellulose-binding domain and glycosyl hydrolase family 61. At the same time, analysis showed a higher number of genes from families related to the degradation of animal material, such as the protein tyrosine kinase and subtilase family. Regarding other protein families, we would like to highlight the high frequency of the LysM domain, with a total of 36 genes, being the largest number of genes reported within the order Onygenales [41,42,43]. The LysM domain is linked to various functions, such as improving fungal–fungal union interactions and chitin and keratin degradation, the latter being fundamental in a keratinophilic fungus.

In recent years, various keratinases have been identified both in bacteria and fungi. In bacteria, these enzymes have been reported in some species of *Bacillus*, *Pseudomonas* and *Stenotrophomonas*, among others, and in fungi in genera such as *Microsporum*, *Onygena* and *Trichophyton* [44]. Keratinases are distributed across various families belonging to the serine proteases and metalloproteases [45]. In the current genome, various families of peptidases that were previously associated with keratin degradation [45,46,47] were identified, such as peptidase family S41, dipeptidyl peptidase IV (DPP IV), peptidase family M16, peptidase family M28, and the fungalysin metallopeptidase (M36), peptidase family M3 and peptidase family M48, which could be linked to the fact that *C. keratinophilum* has been described as a keratinophilic species. In this way, keratin degradation by *C. keratinophilum* could go along the following pathway: a rupture of the keratin disulfide bonds bisulfite reductases; then, the endoproteases of the M36 family would act, providing small peptides; next, exoproteases of the M28 family and dipeptidyl peptidase IV (DPP IV) hydrolyze the peptides into oligopeptides; and, finally, the peptidase M3 family of enzymes can hydrolyze these oligopeptides.

**Figure 3:**
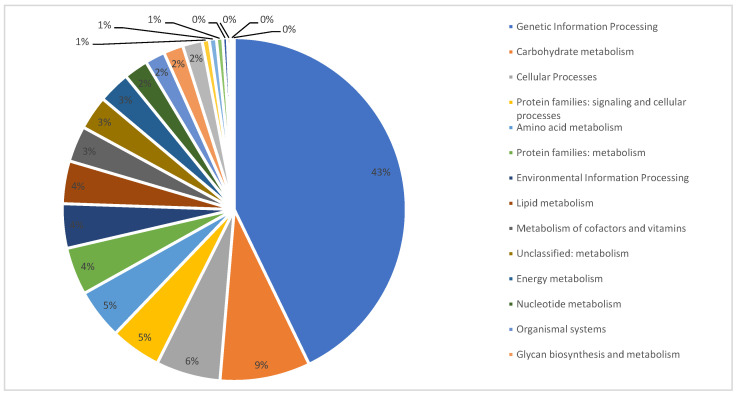
Proportion of genes of *C. keratinophilum* CBS 104.62 assigned to different KEGG functional categories, using BLASTKoala.

The BlastKOALA tool is a KEGG web service that annotates genomes in order to understand the biological functions and interactions of genes [48]. KEGG route-mapping assigned the annotated genes into six levels and distributed them across 22 KEGG categories. Of the six levels, the most prevalent was metabolism (2921, 39.5%), followed by human diseases (1811, 24.5%) and genetic information processing (808, 10.9%). These enzymes were then categorized according to the functional category. The five most prevalent were: genetic information processing (1597, 43%), carbohydrate metabolism (317, 9%), cellular processes (226, 6%), protein families: signaling, cellular processes (178, 5%) and amino acid metabolism (176, 5%) (Figure 3).

The carbohydrate-active enzymes (CAZymes) are a broad class related to the breaking down of complex carbohydrates and polysaccharides into small molecules [49]. Analysis of CAZymes showed that the genome of *C. keratinophilum* encodes a large, varied set of CAZyme families that resulted in the identification of 421 genes (Table 3 and Supplementary Tables S5 and S6), a lower value compared to human pathogenic species of the same order, such as *Blastomyces dermatitidis*, *C. immitis* or *C. posadasii* [50]. Based on the results obtained with DIAMOND, glycoside hydrolases (GHs) were the most prevalent family, with 61 enzymes. The next most prevalent were glycosyltransferases (GTs) with 41, the third was the carbohydrate-binding module (CBM) group with 22, followed by the families of auxiliary activities (AAs), carbohydrate esterase (CE) and polysaccharide lyases (PLs) with 15, 7 and 2, respectively.

The glycosyltransferase enzymes catalyze the formation of glycosidic bonds by the transfer of sugar moieties from activated donor molecules to specific acceptor molecules [51]. In the present study, the most prevalent glycosyltransferases were GT2, GT1 and GT22. The GT2 family was the group with the highest number of genes (with 18), and is one group of enzymes that synthesizes chitin [52]. Previous investigations have shown that the GT2 families are the most common component in most fungal species [51]. The GT1 enzyme encodes sterol glucosyltransferase, which catalyzes the synthesis of sterol glycosides and membrane-bound lipids, and is widespread in some algae, fungi, bacteria, and animals [53]. Finally, the GT22 family is involved in α-1,2-mannosyltransferase activity, which was previously found to contribute to virulence in fungi [54].

The family of glycoside hydrolases (GHs) hydrolyze the glycosidic bond between two or more carbohydrates, or between a carbohydrate and a non-carbohydrate [55]. In the present study GH18, GH47 and GH125 were the most prevalent in this family. The chitinases from family GH18 have been reported previously in fungi and plants. In the case of fungi, these enzymes relate to nutrition, growth, mycoparasitism and virulence [49]. The enzymes GH47 and GH125 relate to the activity of α-mannosidase, although there has been no information on the function of these enzymes until now [56].

For the other families, the most prevalent were CBM50, CE8, AA3 and PL3_2. The CBM50 family is associated with chitinase catalytic domains, implicated in binding chitin [57]. The CE8 family has pectin methylesterase activity, which is essential for the metabolism of pectin [58]. The AA3 family has FAD-dependent (GMC) oxidoreductase activity, relating to the formation of metabolites such as hydroquinones or H_2_O_2_, required by other AA enzymes [59]. Finally, the PL3_2 family is a pectin lyase that catalyzes the scission of pectin [58].

Previous studies performed on different pathogenic fungi genera related to *Chrysosporium*, such as *Blastomyces*, *Coccidioides*, *Histoplasma* and *Sporothrix*, have shown the absence of CAZymes of the PL class [50,60]. In the genome of strain CBS 104.62, the identification of PL3_2 and PL1_7, both related to pectin degradation, was possible [58,61]. Moreover, it was also possible to identify another CAZyme related to pectin hydrolysis, GH28, also absent in the *Coccidioides* genome. The presence of these families in the analyzed genome could be due to the fact that *C. keratinophilum* is a saprophyte fungus with soil as its main ecological niche.

**Figure 4:**
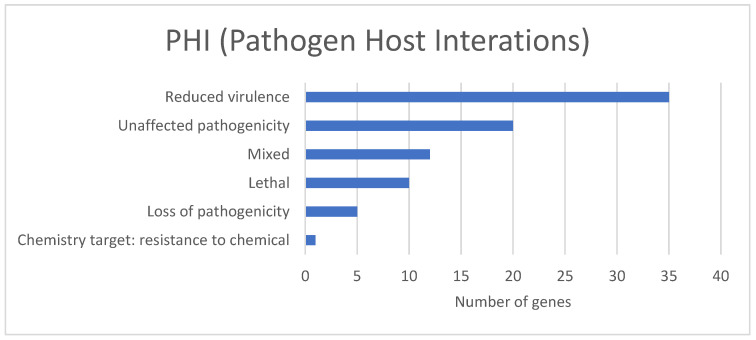
Total of predicted genes of the strain CBS 104.62, that shared significant homology with genes in the pathogen–host interaction (PHI) database.

The PHI base is a database that contains verified information on virulence-related genes that affect the outcome of pathogen–host interactions [36]. Based on the PHI analysis, we identified a total of 83 PHI putative genes in the *C. keratinophilum* genome (1.06% of total genes) (Figure 4 and Supplementary Table S7), *Aspergillus fumigatus* being the species with the highest number of homologous genes (30 genes), followed by *Fusarium graminearum* (20 genes), *Magnaporthe oryzae* (15 genes) and other fungal species (20 genes). Among the genes, the reduced virulence group showed a higher number of genes (35 genes), followed by unaffected pathogenicity with 21, and mixed with 21 genes. The high number of reduced virulence and unaffected pathogenicity can indicate that *C. keratinophilum* CBS 104.62 might be considered to have a weak pathogenic ability. However, various studies consider some strains of *Chrysosporium* spp. as opportunistic pathogens, causing skin and nail diseases, and deeper infections in immunocompromised patients [62].

**Figure 5:**
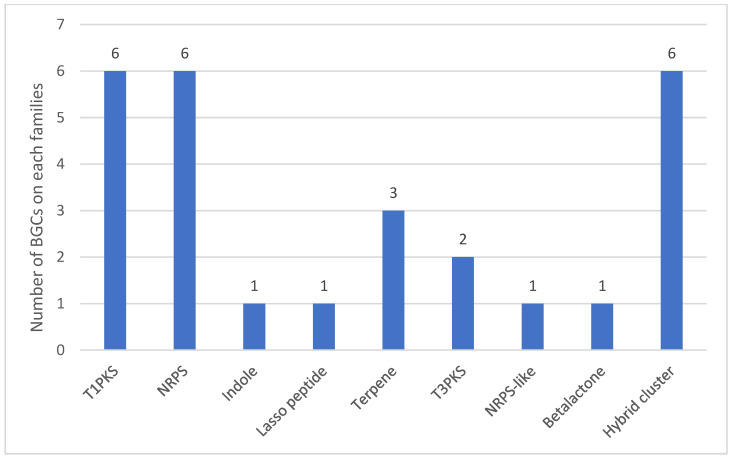
Identified biosynthesis gene clusters (BGCs) in *C. keratinophilum* (CBS 104.62). Abbreviations are as follows: (NRPS) non-ribosomal peptide synthetase clusters, (T1PKS) type 1 polyketide synthase clusters, (T3PKS) type 3 polyketide synthase clusters and (NRPS-like) non-ribosomal peptide synthetase-like cluster.

The secondary metabolite analysis, using AntiSMASH, classified 27 BGCs into nine types, which, according to the genomic organization principle implicated upon transcriptional regulation, could have a role in the production of secondary metabolites by this strain [63] (Figure 5): six non-ribosomal peptide synthetase (NRPS) clusters, six type 1 polyketide synthase (T1PKS) clusters, tree terpene clusters, one indole cluster, two type 3 polyketide synthase (T3PKS) clusters, one lasso peptide, one non-ribosomal peptide synthetase (NRPS)-like cluster, one beta-lactone and six hybrid clusters. From this, one BGC can be identified as okaramine B, with 85% similarity, and the other three as UNII-YC2Q1O94PT YC2Q1O94PT (ACR toxin I), clavaric acid and dimethyl coprogen, with 100% similarity. The UNII-YC2Q1O94PT (ACR toxin I) is associated with the production of leaf spot disease on rough lemon by *Alternaria alternata* [64], clavaric acid is an antitumor isoprenoid compound that acts as an inhibitor of Ras farnesyl transferase, previously described in *Hypholoma sublateritium* [65], and finally, dimethyl coprogen is well known as a siderophore to chelate iron during depleted conditions by *Alternaria alternata* [66].

A previous study determined transcriptionally active genes, as well as their enzymatic products after classifying the biosynthetic genes, using the fungal genomes of anaerobic fungi from the class Neocallimastigomycetes under laboratory conditions [67]. Although our results suggest the probable production of secondary metabolites associated with *C. keratinophilum*, more studies are needed to prove the production of these compounds by this strain.

## 4. Conclusions

In this study, we present the only genome of *Chrysosporium keratinophilum* that has been sequenced and published using a hybrid assembly strategy to date. The genome annotation and the genomic analysis provide new knowledge that will allow us to deepen our understanding of the biology of *Chrysosporium keratinophilum*, and gather new information for further investigations within the Onygenales. In addition, its genetic capability to produce secondary metabolites was successfully determined by the elucidation of the biosynthetic gene pathways, suggesting that the studied strain has a great biosynthetic potential to produce compounds of biotechnological interest. However, future analysis will be necessary to corroborate the in vitro production of such molecules.

## Figures and Tables

**Table 1:** Strains used in the average nucleotide identity (ANI) analysis.

No.	Species	Strain	GenBank Strain BioSample *	GenBank Assembly Accession	Level	Release Date
1	*Amauroascus niger* (asexual morph unknown)	UAMH 3544	SAMN03741936	GCA_001430945.1	Scaffold	22/04/22
2	*Aphanoascus verrucosus* (asexual morph *Chrysosporium tropicum*)	IHEM 4434	SAMN15800566	GCA_014839905.1	Scaffold	22/04/22
3	*Brunneospora queenslandica* (asexual morph *Chrysosporium queenslandicum*)	CBS 280.77	SAMN03741938	GCA_001430955.1	Scaffold	22/04/22
4	*Coccidioides immitis* (asexual morph malbranchea-like)	RS	SAMN02953601	GCA_000149335.2	Scaffold	24/04/22
5	*Coccidioides posadasii* (asexual morph malbranchea-like)	C735 delta SOWgp	SAMN02953748	GCA_000151335.1	Scaffold	24/04/22
6	*Ophidiomyces ophidiicola* (asexual morph chrysosporium-like)	CBS 122913	SAMN23192527	GCA_022830035.1	Scaffold	22/04/22
7	*Uncinocarpus reesii* (asexual morph unknown)	UAMH 1704	SAMN02953631	GCA_000003515.2	Scaffold	22/04/22

CBS = Westerdijk Fungal Biodiversity Institute fungal and yeast collection, Utrecht, The Netherlands; IHEM = BCCM/IHEM fungi collection: human and animal health, Brussels, Belgium; UAMH = UAMH Centre for Global Microfungal Biodiversity, Toronto, ON, Canada; C735 delta SOWgp; and RS strains = Broad Institute, Cambridge, MA, USA. * BioSmaple (https://www.ncbi.nlm.nih.gov/biosample/ (accessed on 22 April 2022)).

**Table 2:** Genome assembly statistics of *Chrysosporium keratinophilum* and phylogenetically related onygenalean taxa.

Genome Statistics	*Chrysosporium* *keratinophilum* CBS 104.62^T^	*Aphanoascus verrucosus* (Asexual Morph *Chrysosporium tropicum*) IHEM 4434^T^	*Brunneospora queenslandica* (Asexual Morph *Chrysosporium queenslandicum*) CBS 280.77^T^	*Ophidiomyces ophidiicola* (Asexual Morph Chrysosporium-like) CBS 122913^T^	*Uncinocarpus reesii* (Asexual Morph Unknown) UAMH 1704	*Coccidioides immitis* (Asexual Morph Malbranchea-like) RS	*Coccidioides posadasii* (Asexual Morph Malbranchea-like) C735 Delta SOWgp
Contigs (≥0 bp)	25	211	2724	116	45	7	55
Total length (≥0 bp)	25,439,844	23,059,040	32,335,957	21,970,319	22,349,738	29,016,019	27,013,412
Largest contig (bp)	5,001,415	894,230	979,930	1,803,704	7,891,746	8,482,323	5,398,309
G+C content (%)	49.09	49.59	53.15	47.64	48.66	45.96	46.59
N50 (bp)	2,037,736	431,852	173,991	506,472	5,332,914	4,323,945	2,376,830
N90 (bp)	460,884	132,993	4367	122,144	2,507,206	3,458,857	974,251
L50	4	17	47	12	2	3	4
L90	14	52	798	42	5	6	11
# N’s per 100 kbp	10.06	10.51	6001.89	1.81	812.87	1.38	0.12

**Table 3:** Carbohydrate-active enzyme (CAZyme) content in the *C. keratinophilum* genome.

Carbohydrate-Active Enzyme (CAZyme) Classes	Number of Identified Families	Number of Identified Genes
Glycoside hydrolases (GHs)	61	156
Glycosyltransferases (GTs)	41	151
Carbohydrate-binding module (CBM)	22	57
Auxiliary activities (AAs)	15	36
Carbohydrate esterase (CE)	7	18
Polysaccharide lyases (PLs)	2	3

## Data Availability

This Whole Genome Shotgun project has been deposited in DDBJ/ENA/GenBank, under the accession JARBIX000000000; BioSample SAMN33269181.

## Supplementary Materials

The following supporting information can be downloaded at: https://www.mdpi.com/article/10.3390/jof9040389/s1, Table S1: Identified PFAMs in the *C. keratinophilum* CBS 104.62 genome; Table S2: Summary of PFAMs in the *C. keratinophilum* CBS 104.62 genome; Table S3: Identified superfamily in the *C. keratinophilum* CBS 104.62 genome; Table S4: Summary of superfamily in the *C. keratinophilum* CBS 104.62 genome; Table S5: Identified CAZymes in the *C. keratinophilum* CBS 104.62 genome; Table S6: Identified CAZymes in the *C. keratinophilum* CBS 104.62 genome for each family; Table S7: Identified PHIs in the *C. keratinophilum* CBS 104.62 genome.
